# Custom-Shaped Organic Photovoltaic Modules—Freedom of Design by Printing

**DOI:** 10.1186/s11671-017-1871-9

**Published:** 2017-02-14

**Authors:** M. Välimäki, E. Jansson, P. Korhonen, A. Peltoniemi, S. Rousu

**Affiliations:** VTT Technical Research Centre of Finland Ltd, Oulu, Finland

**Keywords:** Printed OPV, Ultra-thin and lightweight, Custom-shaped OPV, Arbitrary shape OPV, Freedom of design

## Abstract

Freedom of design that was introduced as organic photovoltaic (OPV) modules were fabricated by printing. As proof-of-concept, we show OPV leaf fabrication in A5 size using gravure and rotary screen printing processes for the main active layers of the OPV structure. These printing methods allow direct printing of any kind of arbitrary, two-dimensional shapes including patterning of the electric contacts thus post-patterning stages are not needed. Fabrication of custom-shaped OPV modules requires detailed information about the technical boundaries set by the manufacturing process and materials which in turn influence the layout design and R2R upscaling. In this paper, we show custom-shaped OPV modules, patterned directly in a shape of a tree leaf with an overall size of 110 cm^2^ and an active area of 50 cm^2^ providing a power conversion efficiency of 2.0% and maximum power of 98 mW.

## Background

Thin-film PV technologies have huge potential to spread into a wide range of off-grid end-use areas where autonomous energy harvesting is needed [1]. In the case of organic photovoltaics (OPV), flexible, lightweight and ultra-thin device stack can be obtained using well-established and widely used roll-to-roll (R2R) mass-production methods under ambient atmosphere [2]. OPV modules have been fabricated using various R2R compatible printing [3–9] and coating techniques [10–13]; however, the use of coating methods limits the ability to directly pattern arbitrary, custom-shaped two-dimensional features [14, 15] which is considered to be a key factor for the successful commercialization of OPVs as recently recognized by many commercial players although technical data concerning the functionality is not available. In effect, customization of OPV is the key advantage of using printing over coating thus unlimited designs to any two-dimensional shape is possible, giving true design freedom for OPVs and electrical connections in the modules. Design of freedom is especially important in building integrated and interior energy harvesting applications [16]. Currently, there are only few papers dealing with truly two-dimensional-patterned OPV modules. Eggenhuisen et al. fabricated ink-jet printed single solar cells in the shape of “Christmas tree”, and Krebs et al. were the first ones to show screen printed “Solar hat”-module although the electrical performance of both shapes was poor/limited [4, 17].

In this paper, we demonstrate freedom of design by manufacturing OPV modules in the shape of a “tree leaf” which provide good electrical properties. As proof-of-concept, we show OPV leaf fabrication in A5 size using gravure and rotary screen printing processes for the main active layers of the OPV structure. These printing methods allow direct printing of any kind of arbitrary, two-dimensional shapes including patterning of the electric contacts and thus no post-patterning stages are needed. Successful fabrication of custom-shaped OPV modules requires detailed information about the technical boundaries set by the manufacturing process and materials which in turn influence the layout design. The used printing processes are also R2R up-scalable [8, 9]. R2R upscaling is demonstrated in this paper although further optimization is a subject for the future. In this work, we evaluate the technical aspects affecting the design freedom, and as an outcome fabricate OPV leaves with an overall size of 110 cm^2^ and an active area of 50 cm^2^ [18].

## Results and Discussion

The custom-shaped OPVs were fabricated using a structure of five layers in which the first three layers were patterned using screen printing and gravure printing techniques. The processing comprised the patterning of indium-tin-oxide (ITO), printing of poly (3,4- ethylenedioxythiophene):poly (styrenesulfonate) (PEDOT:PSS) and poly(3-hexylthiophene-2,5-diyl):[6,6]-phenyl C61 butyric acid methyl ester (P3HT:PCBM) layers and thermal evaporation of lithium fluorine (LiF) and aluminium (Al) through a shadow mask to demonstrate the proof-of-concept. However, the evaporation phases can be replaced with wet deposition methods such as printing especially when an inverted device stack is used. In that case, for instance PEDOT:PSS and silver inks can be printed as hole transport layer and hole contact layers using screen printing and flexography printing [7, 9, 19]. Here, sputtered ITO was patterned in R2R rotary screen printing process, and PEDOT:PSS and P3HT:PCBM were gravure printed using lab-scale printer. Currently, various suitable electrode and interlayer materials have been introduced for wet-chemical processing [19]. Furthermore, the alternative active materials have reached efficiencies close to 11% [20]. The record cells with novel active materials are typically small and fabricated with non-scalable processing techniques under inert conditions whereas P3HT:PCBM-based modules have been widely processed under ambient conditions [19]. However, some materials that are scalable and suitable for wet-deposition under ambient air might still be prone to cause reduction of efficiency if for instance the desired layer thickness/morphology is difficult to produce in a reproducible manner. Successful demonstration made by Berny et al. shows the reduction of efficiencies from 9.3 to 4.5% when the substrate, electrode and interlayer materials were replaced with the materials that are scalable [16]. From the fabrication point of view, it is also crucial that materials obtain uniform quality, sufficient stability and availability in larger quantities for the optimization of the printing inks and processes. Detailed information concerning the materials, equipment and processing used to demonstrate the proof-of-concept for custom-shaped free-form OPVs is described in “Methods Section”.

### Custom-Shaped OPVs: the Boundary Conditions for Freedom of Design

The entire OPV device stack can be directly printed into any kind of two-dimensional shape using mass-manufacturing printing technologies, namely gravure, flexography and screen printing [2, 13]. These printing methods and the materials used for printing however set some technical boundary conditions for the layout design, which are especially important for the electrical performance of the modules. In this work, the technical boundaries for the custom-shaped OPVs were determined by the processing method, the used equipment and materials. First, the overall feature size was defined to be 120 by 130 mm. This was limited by the engraved area of the gravure printing plate and the size of the evaporation mask. Secondly, the dimensions of the individual OPV cells were influenced by the electrode materials. The length of the cell was limited to 50 mm due to the thermal evaporating process of LiF/Al where the fine features of the shadow mask are prone for bending, and the width of the cells were fixed into 5 mm due to the sheet resistance of ITO [5]. Additionally, the precision of the printed image is influenced by the dimensional accuracy of the printed layer i.e. the shrinking/spreading of the ink thus the dimensions of the test prints were compared with the layout. In R2R rotary screen printing of ITO, the dimensions in machine (MD) and cross-machine direction (CD) perpendicular to the direction of printing deviated +0.2% (MD) and +0.1% (CD). In S2S, gravure printing of PEDOT:PSS dimensions deviated +4% (MD) and +0.4% (CD) compared to the layout and respectively, P3HT:PCBM −0.9% and +0.1%.

The accuracy of alignment was defined by the gravure printer since the alignment was made manually without any additional registration system. The arbitrary shaped OPV cells have higher requirements in the dimensional accuracy and with the layer alignment compared to rectangular cells. All layers of arbitrary shaped cells are dependent on both machine direction (MD) and cross-machine direction (CD) whereas rectangular shape cells can be partially fabricated in the form of the stripe. Moreover, MD misalignment will decrease the tolerance of CD alignment and vice versa thus, in practice the tolerances can be reduced into half. This means that any complexity in the geometry will increase the dependency of the alignment between machine and cross-machine direction which in turn, will reduce the tolerance of the alignment. The tolerance covering the influences caused by the misalignment and dimensional variations of the printed pattern due to spreading or shrinking was designed to allow the deviation of 300 μm in both directions between ITO-PEDOT:PSS and ITO-P3HT:PCBM. However, the maximum tolerance of 300 μm can exist only with perfect orthogonal alignment although in practice some deviation on the other direction always exists. Additionally, the tolerance of the alignment between the ITO layer and the shadow mask for the cathode evaporation was set to allow the deviation of 150 μm.

**Fig. 1:**
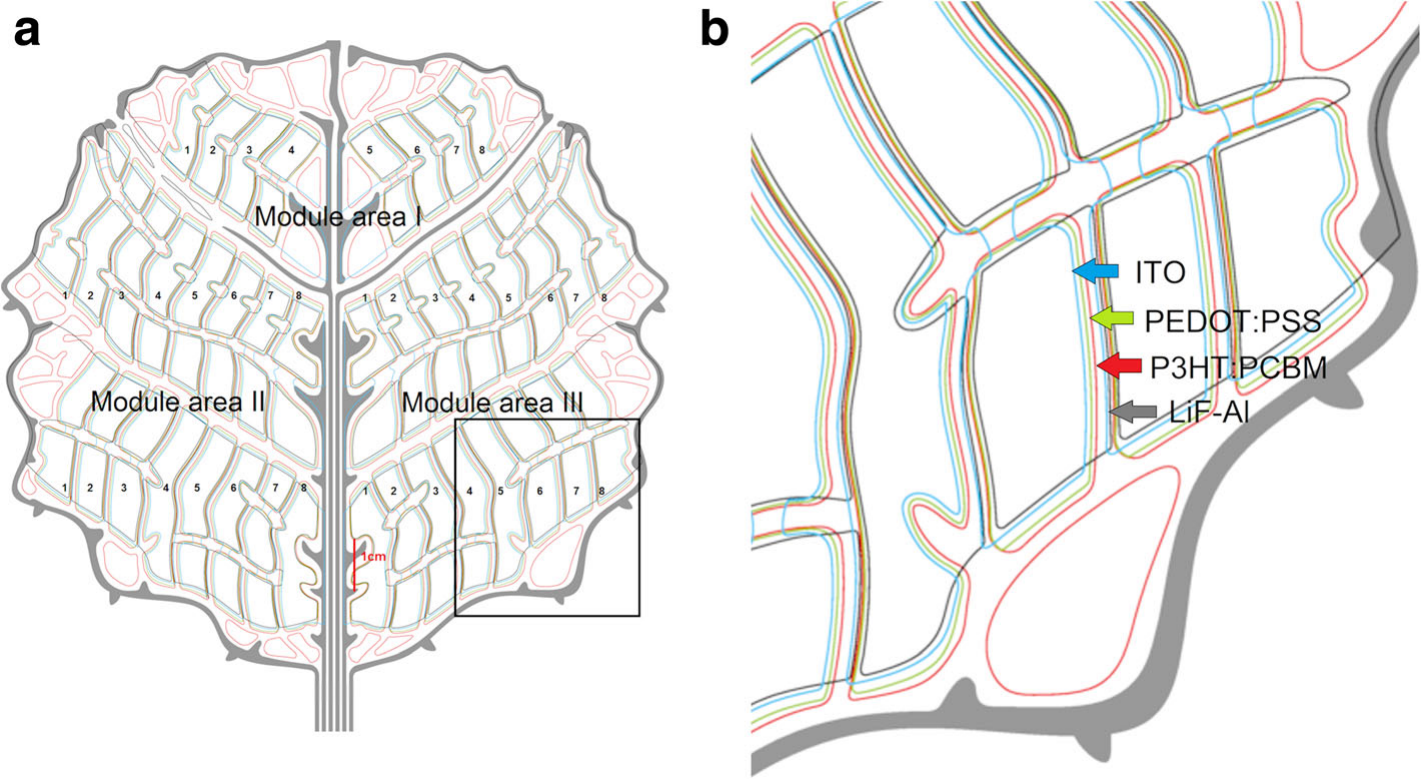
Custom OPV shapes fabricated in a form of tree leaf; the layout (**a**) comprises of three modules with active area of 7.5 cm^2^ (area I) and 21.8 cm^2^ (areas II and III). Furthermore, serially connected OPV cells are numbered from one to eight. The magnified image (**b**) shows the alignment from the left as follows: ITO, PEDOT:PSS, P3HT:PCBM and LiF-Al

The custom OPV shapes presented in Fig. 1 were designed to form a leaf of tree comprising three modules with the active area of 7.5 and 21.8 cm^2^. Cells with the active area of 0.94 cm^2^ (smaller modules) and 1.36 cm^2^ (larger modules) were monolithically connected together by overlapping eight cells in series. Furthermore, the larger modules comprised of two sets of eight serially connected cells which were further connected together in parallel.

### Alignment of Custom-Shaped OPVs

**Fig. 2:**
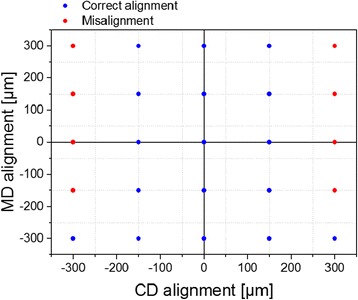
Operating window for the deviation of machine (MD) and cross-machine (CD) alignment in gravure printing of OPV leaves

**Fig. 3:**
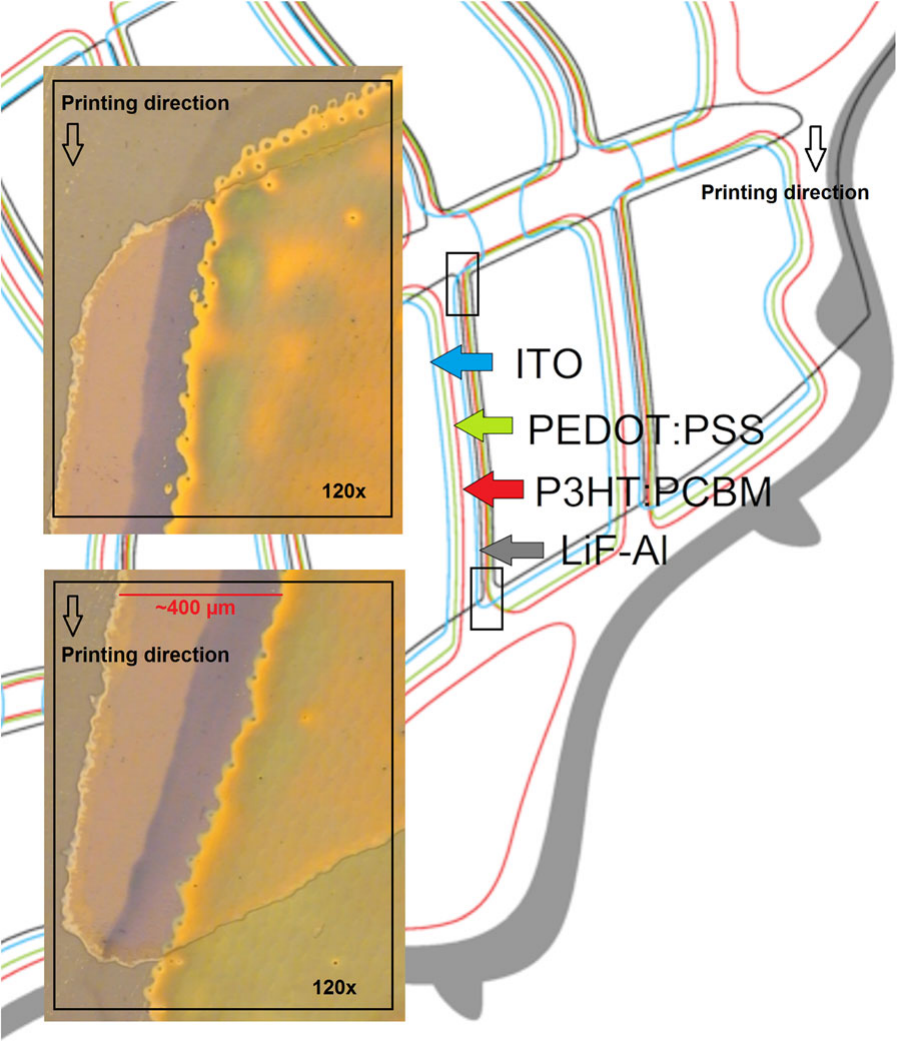
Microscopic images presenting the gravure printed PEDOT:PSS and P3HT:PCBM layers and their alignment on top of patterned ITO

Since there is a dependency between the MD and CD alignment, the tolerance of the alignment in printing was evaluated in both directions simultaneously and depicted using a window of operation in Fig. 2. As the graph shows, the custom OPV shapes forming a leaf allow CD tolerances of ±150 μm and MD of ±300 μm. In addition, higher CD tolerances are allowed as the printed image is forwarded and the MD alignment is shifted from zero to the negative. Additionally, the CD alignment of the layout is more prone for deviation as the sides of the leaf are forming mirror images. This means that misaligned layer can cover the bottom electrode contacts on the other side of the leaf preventing the serial connection between the cells, and short-circuit the cells on the other side of the leaf if the photoactive layer is not covering the entire cell area. Figure 3 presents the alignment accuracy of gravure printed PEDOT:PSS and P3HT:PCBM layers on top of patterned ITO. Furthermore, the microscopic images show the uniformity and patternability of printed layers. The comparison between the design layout and the microscopic images shows the acceptable alignment between PEDOT:PSS, P3HT:PCBM and ITO layer. Alignment of gravure printed PEDOT:PSS layer with ITO was forwarded in the printing direction (MD) whereas gravure printed P3HT:PCBM layer with ITO was slightly delayed and shifted to the opposite direction. The cross-machine alignment of PEDOT:PSS and P3HT:PCBM layers were shifted closer to the edge of ITO on the left side of the leaf although the deviations were within the specified tolerance range providing an acceptable alignment.

### Electrical Performance of OPV Leaf Modules

**Fig. 4:**
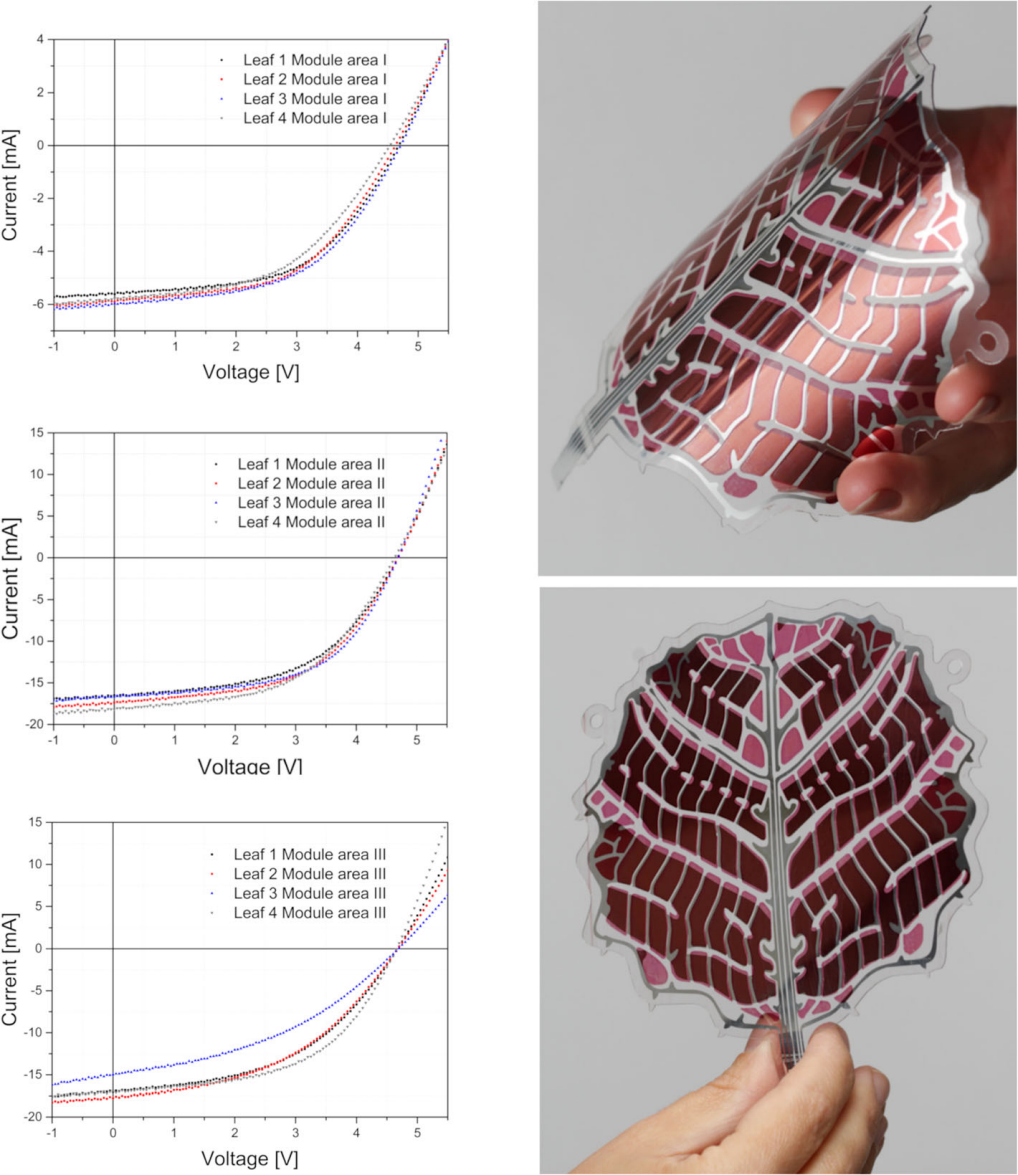
IV-characteristics and photographs of OPV leaf modules. Areas I–III represent the different module areas (area I: *upper leaf area* with the size of 7.5 cm^2^, areas II and II both with the size of 21.8 cm^2^ located *under the upper leaf module*) of four individual OPV leaves

OPV leaf was divided into three modules where one 7.5 cm^2^ module (area I) was located above two 21.8 cm^2^ modules (area II and area III). All module areas comprised of eight serially connected cells obtaining an open circuit voltage (V_oc_) of 4.7 V for the area I module and 4.7 V for areas II and III modules. An average V_oc_ of 0.58 V per cell indicate good quality of printed layers. In area I, the eight serially connected cells resulted in a short-circuit current (I_sc_) of 5.9 mA whereas areas II and III modules comprised parallel connection between two sets of eight serially connected cells and resulted in I_sc_ of 18 mA. Measurements were made with AM 1.5 solar simulator under the illumination of 1 sun (1000 W/m^2^), without any mismatch correction for the spectral response. Maximum power conversion efficiencies (PCE) of 1.9–2.0% were obtained in all areas of the OPV leaf, and in the maximum power point, OPV leaf can provide power ranging from 87 to 98 mW. The electrical parameters of all OPV leaf modules are summarized in Table 1 and the IV-characteristics of OPV leaf modules are presented in Fig. 4 together with the photographic images of the OPV leaf. The electrical properties of the free-form modules are parallel with the results that have been reported when similar device structure is fabricated using a rectangular design where the module is formed by striped cells [8, 19]. However, it is likely that the resistance of electrode material i.e. ITO on top of PET substrate is limiting the efficiency thus, the use of alternative electrode material could improve the efficiency or the fabrication of smaller cell size when ITO is used as electrode material [5, 19].

**Table 1:** Electrical parameters of OPV leaf modules (with separate areas I–III) illuminated under 1 sun (AM 1.5)

OPV leaf	Leaf area	Active area [mm^2^]	I_sc_ [mA]	V_oc_ [V]	V_oc_/cell [V]	FF [%]	PCE [%]	P_max_ [mW]
Leaf 1	Area I	748	5.6	4.7	0.59	53	1.9	14
	Area II	2180	16	4.7	0.59	52	1.9	40
	Area III	2180	17	4.7	0.58	47	1.7	38
Leaf 2	Area I	748	5.9	4.6	0.58	52	1.9	14
	Area II	2180	17	4.7	0.59	53	2.0	43
	Area III	2180	18	4.7	0.58	45	1.7	37
Leaf 3	Area I	748	6.0	4.7	0.59	52	2.0	15
	Area II	2180	17	4.7	0.59	56	2.0	44
	Area III	2180	15	4.7	0.59	40	1.3	28
Leaf 4	Area I	748	5.8	4.5	0.57	49	1.7	13
	Area II	2180	18	4.6	0.58	51	2.0	43
	Area III	2180	17	4.7	0.58	53	1.9	42

**Fig. 5:**
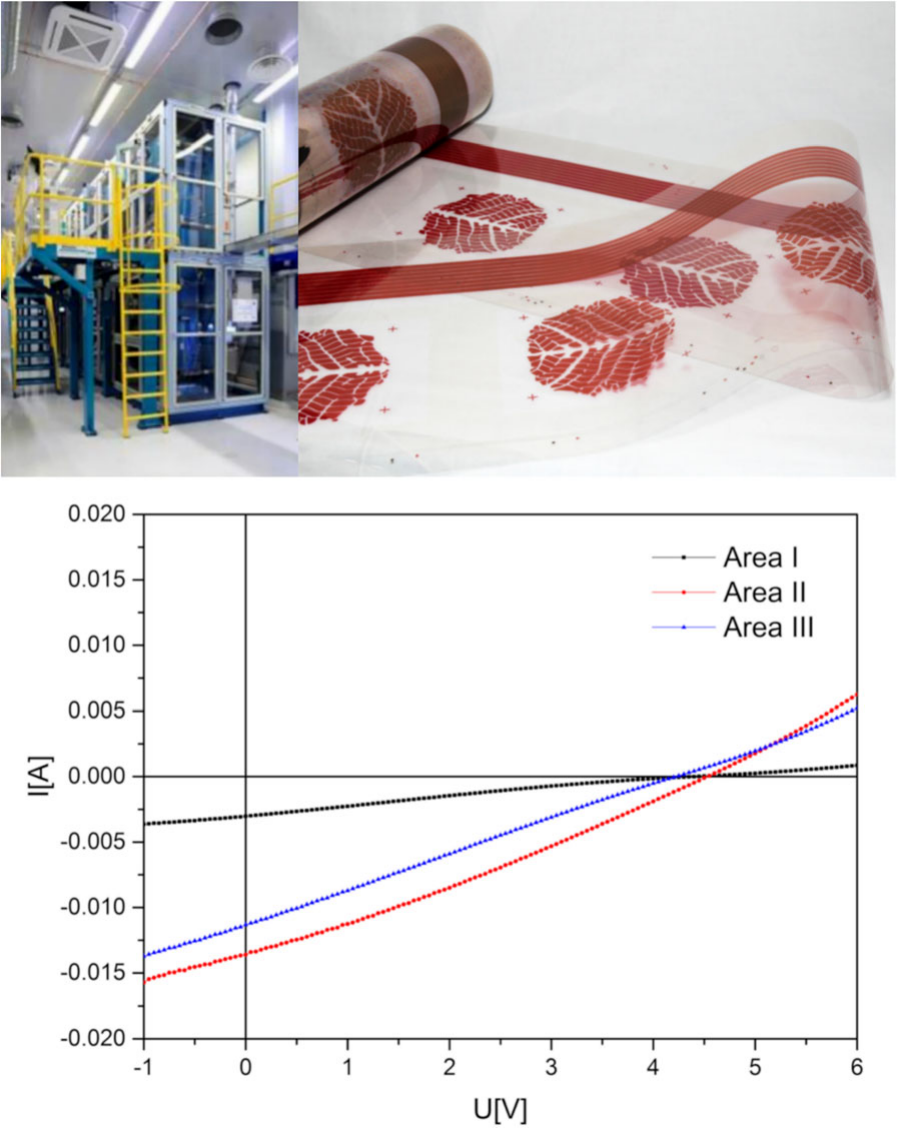
IV-characteristics of custom-shaped OPV leaf modules fabricated using R2R-printing machine

Here, the potential of custom-shaped OPV has been demonstrated with sheet-based printing equipment. Furthermore, the first demonstration for roll-to-roll upscaling of PEDOT:PSS and P3HT:PCBM printing for OPV leaf resulted V_oc_ values up to 4.5 V and I_sc_ values up to 13.6 mA. The produced modules were functional although the efficiencies did not exceed above 0.8% (Fig. 5). The lower efficiency was caused by the lack of optimization of the material formulations and processing parameters (e.g. speed and drying) for the continuous process. The materials and processing parameters used in roll-to-roll environment are typically different from the materials and parameters used in sheet-fed processes resulting from the different ink flow and ink transfer mechanisms, as well as the larger amount of ink in the process especially when high demands for the accuracy of registration and layer quality are required. In addition, the constantly moving web during the roll-to-roll process is limiting the drying profile and in contact with several (process) cylinders that increases the effect of the static electricity on the device performance. Thus, to obtain highly functional R2R processed OPVs with free-form design, the upscaling needs further optimization.

## Conclusions

Freedom of design that was introduced as organic photovoltaic (OPV) modules were fabricated by printing. These custom-shaped OPV modules were directly patterned in a shape of tree leaf using gravure printing and rotary screen printing processes, allowing direct printing of any kind of arbitrary, two-dimensional shapes. The fabrication of custom-shaped OPVs produced information concerning the technical boundaries of layout design and manufacturing prior to further steps towards more complex OPV designs, module characterization and process upscaling are taken. As an outcome of the work, we fabricated OPV leaves with an overall size of 110 cm^2^ and an active area of 50 cm^2^ achieving a power conversion efficiency of 2.0% and maximum power of 98 mW.

### Methods

The solar cell stack was prepared on ITO-PET substrate OC-50 from Solutia providing sheet resistance of 50Ω/□. ITO layer was patterned in R2R rotary screen printing process using HiperEtch etching paste purchased from Merck [21]. The patterned ITO-PET roll was processed further with sheet-to-sheet equipment. First, a 30-nm layer of PEDOT:PSS (Clevios VP AI 4083 purchased from Heraeus) was gravure printed as a hole transporting layer on top of ITO layer with lab-scale printer (Labratester, Norbert Schläfli Maschinen). PEDOT:PSS ink was prepared by mixing the PEDOT:PSS and isopropyl alcohol with a ratio of 77:23 wt% and a line density of 140 lines cm^−1^: In order to enable the patterning of low viscous PEDOT:PSS ink into the shape of the leaf without excessive ink spreading, the amount of isopropyl alcohol and size of the engraving were minimized.

PEDOT:PSS was printed with a speed of 7 m/min and left overnight at +80 °C. Respectively, 200-nm-thick photoactive layer of P3HT:PCBM blend was gravure printed on top of PEDOT:PSS with lab-scale printer (Labratester, Norbert Schläfli Maschinen) using a printing speed of 7 m/min, a line density of 120 lines cm^−1^ and dried slowly in a covered box before the hot plate treatment under nitrogen at +120 °C for 10 min. Regioregular P3HT (#4002-E, Rieke Metals) was used as the donor and PCBM [C60] (purity 99.5%, Nano-C) as the acceptor in the photoactive blend which was dissolved in 1,2-dichlorobenzene to obtain solid concentration of 0.13 g ml^−1^. Finally, 1-nm layer of lithium fluorine and 100-nm layer of aluminium were thermally evaporated as a top electrode.
